# Role of Pea Enation Mosaic Virus Coat Protein in the Host Plant and Aphid Vector

**DOI:** 10.3390/v8110312

**Published:** 2016-11-18

**Authors:** Juliette Doumayrou, Melissa Sheber, Bryony C. Bonning, W. Allen Miller

**Affiliations:** 1 Department of Plant Pathology & Microbiology, 351 Bessey Hall, Iowa State University, Ames, IA 50011, USA; mksheber@iastate.edu (M.S.); wamiller@iastate.edu (W.A.M.); 2 Department of Entomology, 339 Science II, Iowa State University, Ames, IA 50011, USA; bbonning@iastate.edu

**Keywords:** Pea enation mosaic virus, coat protein, viral accumulation, viral encapsidation, aphid transmission

## Abstract

Understanding the molecular mechanisms involved in plant virus–vector interactions is essential for the development of effective control measures for aphid-vectored epidemic plant diseases. The coat proteins (CP) are the main component of the viral capsids, and they are implicated in practically every stage of the viral infection cycle. *Pea enation mosaic virus* 1 (PEMV1, *Enamovirus*, *Luteoviridae*) and *Pea enation mosaic virus* 2 (PEMV2, *Umbravirus*, *Tombusviridae*) are two RNA viruses in an obligate symbiosis causing the pea enation mosaic disease. Sixteen mutant viruses were generated with mutations in different domains of the CP to evaluate the role of specific amino acids in viral replication, virion assembly, long-distance movement in *Pisum sativum*, and aphid transmission. Twelve mutant viruses were unable to assemble but were able to replicate in inoculated leaves, move long-distance, and express the CP in newly infected leaves. Four mutant viruses produced virions, but three were not transmissible by the pea aphid, *Acyrthosiphon pisum*. Three-dimensional modeling of the PEMV CP, combined with biological assays for virion assembly and aphid transmission, allowed for a model of the assembly of PEMV coat protein subunits.

## 1. Introduction

As obligate parasites of sessile organisms, the transmission of plant viruses from one host to another is a vital step in their life cycle. While around 80% of plant viruses depend on insect vectors for transmission, more than 70% of all known insect-borne viruses are transmissible by hemipterans [1]. Plant viruses have various transmission strategies involving very specific interactions with their insect vector (i.e., circulative and non-circulative modes [2]). Elucidation of the molecular mechanisms involved in these specialized interactions allows for new approaches to the control of virus diseases that cause extensive agricultural damage. The coat proteins (CPs) are the primary components of the plant virus virion and are implicated in practically every stage of the viral infection cycle [3]. The CP plays roles in viral replication, translation, and movement as well as modulation of host responses to viral infection. The assembly of the CP to form virions is essential for vector-borne transmission. CP is also important for the specificity of transmission, i.e., determining which aphid vectors are able to transmit the virus [4].

Luteoviruses (*Luteoviridae* family) are transmitted by their aphid vectors in a circulative, non-propagative manner. The plant virus is taken up from the aphid gut into the gut epithelial cells and transported to the hemolymph by receptor-mediated transcytosis [5,6]. Virions are carried in the hemolymph to the accessory salivary gland and are released with salivary secretions into the phloem. Pea enation mosaic is a disease of pea (*Pisum sativum* L.) caused by two obligatory symbiotic viral genomes: *Pea enation mosaic virus* 1 (PEMV1), of genus *Enamovirus* (*Luteoviridae*, [7]); and PEMV2, belonging to genus *Umbravirus* (*Tombusviridae*) [8]. Each umbravirus depends on one particular polerovirus or enamovirus for virion assembly and thus transmission by aphids [9]. Therefore, PEMV2 uses the CP encoded by PEMV1 for encapsidation and aphid transmission [8,10]. PEMV is transmitted by several aphid species, *Acyrthosyphon pisum* being one of the most common [11,12]. The PEMV1 CP binds membrane alanyl aminopeptidase N in the gut of *A. pisum*, which has been identified as a receptor for uptake of PEMV required for circulative transmission [6]. However, PEMV1 requires PEMV2 movement protein for long-distance movement in plants [13,14].

The use of PEMV as a model virus has the unique advantage that it is mechanically transmissible, unlike other members of the *Luteoviridae* family. Moreover, the accumulation and spread of PEMV are not limited to the phloem, allowing for high virus titers in infected plants [13,14]. Finally, a specific plant virus receptor that binds PEMV in the gut has been identified in *A. pisum* [6].

**Figure 1:**
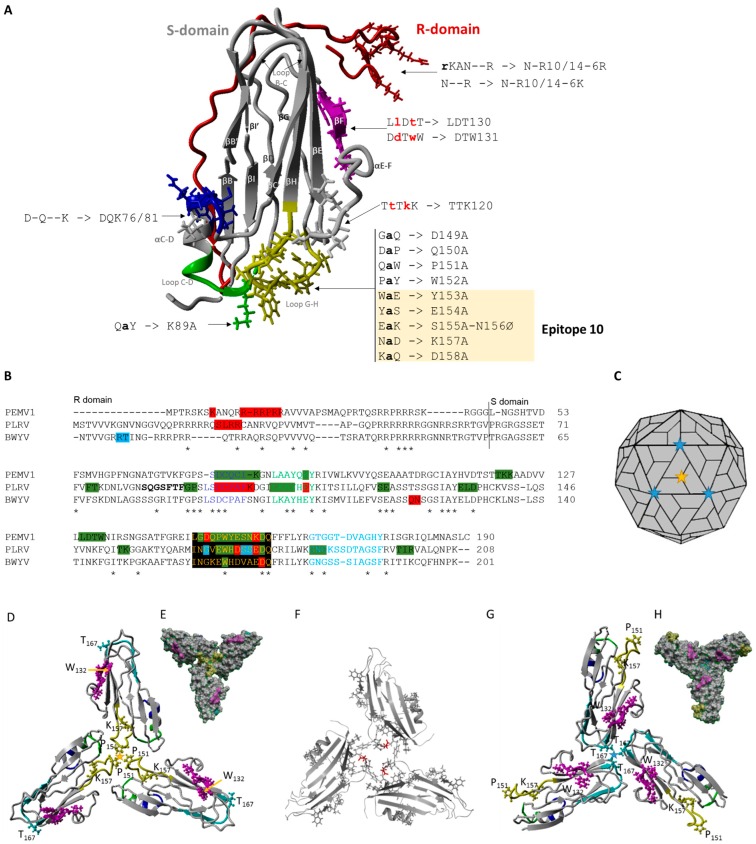
Predicted three-dimensional (3D) structure of *Pea enation mosaic virus* (PEMV) coat protein with the location of the 16 mutations indicated and its predicted structure in the virion. (**A**) The R-domain is shown in red and the S-domain in other colors. The positions of residues that form epitope 10 in the loop regions between strands G and H in the S-domain are shown in yellow. Two surface domains between strands C and D are shown in blue and green [21]. The “hot spot” for virus–host protein interactions is shown in purple [24]. The location of the 16 mutations along with the specific mutation made is indicated as follows: gray lowercase letters indicate the replacement amino acid; blue lower case letters indicate the insertion of a new amino acid; a dash indicates a deletion. (**B**) Alignment of PEMV, *Potato leafroll virus* (PLRV), and *Beet western yellows virus* (BWYV) coat protein sequences. Epitope 10 (black box with yellow letters), two regions predicted to be exposed on the virion surface (dark blue and green letters), and the peptide on the external surface as described by Chavez et al. [23] for PLRV (light blue letters) are shown. Amino acids (aa) implicated in virion assembly (dark green highlights), transmission by aphids (red highlights), and long-distance movement in the plant (blue highlight) are indicated. Asterisks show conserved aa. P_151_ involved in interaction between coat protein (CP) in the trimer is indicated by a yellow star; T_167_, predicted to be important for interaction between CP on the β-annulus, is indicated by a blue star. (**C**) Schematic representation of the location of P_151_ and T_167_ on the *T* = 3 icosahedral virion structure of PEMV; (**D**) predicted model of the PEMV CP trimer viewed from the exterior and (**E**) its representation on the surface. Color code of aa as for (A) Purple indicates the “hot spot” for virus–host protein interactions [24]; (**F**) predicted CP trimer of the mutant P151A; (**G**) the reconstituted trimer of PEMV via the β-annulus and (**H**) its representation on the virion surface. CP model was generated using I-TASSER and the trimers models using SymmDock.

The icosahedral 28 nm diameter PEMV1 virion contains a 5.7 kb positive-sense, single-stranded RNA (+ssRNA) genome while the 25 nm PEMV2, contains a 4.2 kb viral +ssRNA. Virions have a *T* = 3 icosahedral symmetry containing 180 and 140–150 protein subunits for PEMV1 and PEMV2, respectively [15]. PEMV is assembled with subunits of CP trimers encoded by PEMV1 [16]. Virions also contain a minor component of 54 kDa (readthrough protein, RTD) translated when the stop codon of the CP open reading frame (ORF) is suppressed. The RTD is predicted to be exposed on the surface of the virions, and Demler and co-workers [16] showed that RTD, while not required for virion formation, is required for aphid transmission. Unfortunately, no crystallographic data for virions are available for any members of the *Luteoviridae* family. However, the secondary and tertiary CP structures of all icosahedral virions are conserved [17]. The CP can be divided into two domains: (i) the N-terminal arginine-rich domain (named R-domain), which is likely internal and interacts with viral RNA; and (ii) the shell domain (S-domain) conserved among all RNA viruses and forming the core of the virion. The three-dimensional (3D) structure of this domain has a jelly roll configuration formed by an eight-stranded β-barrel [18]. Because *Luteoviridae* is divided into three genera, *Luteovirus*, *Polerovirus*, and *Enamovirus*, they share phylogenetic similarities and distinctive structural features in their genome organization. One of the genes conserved in all luteovirids is the major coat protein. Information about the structure of the luteovirus CP is available for two independent polerovirus models, *Beet western yellows virus* (BWYV) [19] and *Potato leafroll virus* (PLRV; [19,20]). The authors reported amino acids (aa) in domains exposed on the surface of virions [20], mainly in acidic patches, which are involved in different steps of the viral life cycle such as virion assembly, replication and long-distance movement in the plant, and transmission by aphids [19,21,22,23,24]. These domains are located on important antigenic sites in PLRV and *Barley yellow dwarf virus* (BYDV), known as epitope 10 on the loop between β-strands G and H [25]. Epitope 10 contains several aa conserved in most members of the *Luteoviridae* [26] and the residues HDSSEDQ and HDVAEDQ of this epitope 10 were determined to be exposed at the surface of PLRV and BWYV virion, respectively [19,25]. A second domain, close to the middle of the trimer for virion assembly, is located upstream of epitope 10 and is also highly conserved among *Luteoviridae* members (e.g., LKAYHEY in PLRV CP; [20]). Finally, other domains were predicted to be exposed on the surface of virions, conserved between polerovirus CP sequences (PLRV and BWYV) or located in other epitopes, altering the virion stability and/or vector transmission (see Figure 1B; [22,25,26]).

An understanding of the structure of the PEMV virion is essential for understanding the specific molecular interactions between the virion and the aphid vector. The goal of this study is to predict the 3D structure of the CP of PEMV1, and to identify certain aa probably exposed on the surface of the virions that are implicated in viral assembly, replication, and long-distance movement in the pea plant as well as in transmission by aphids. We generated 16 CP mutants that were able to replicate in inoculated leaves, move within the plant, and replicate in upper leaves. Only four of the mutants assembled into virions, one of which was transmissible by aphids. Our results differ from the two polerovirus models, perhaps due to the synergy between two viruses to induce this disease.

## 2. Materials and Methods

### 2.1. CP Predicted Structure

To localize specific sites of the PEMV CP potentially involved in vector-borne transmission, the structure was predicted for the coat protein sequence of PEMV1 accession no. NC_003629 using I-TASSER (http://zhang.bioinformatics.ku.edu/I-TASSER/; [27]). I-TASSER uses a protein structure modeling approach based on multiple threading alignments and iteration to obtain the five most likely structures related to the native structure based on confidence scores (C-score). The C-score indicates the best estimated model based on the global accuracy of models, where a higher value signifies a model with high confidence. The template modeling TM-score and root-mean-square deviation (RMSD) are standards used for measuring structural similarity between two structures. A correct topology of the predicted protein structure is indicated by a TM-score >0.5, while a TM-score of <0.17 implies random similarity. The estimation of the residue-specific quality (RSQ) by ResQ [28], using a combination of both template-based assignment and profile-based prediction, was used to support the residue-level quality of the protein structure. A low-quality prediction of the model structure has relatively higher modeling error shown by high estimated distance values (in angstroms). The predicted structure of PLRV CP [21,22,23,24] was used as a reference for this study. The structure predictions of PEMV1 CP were visualized using YASARA (www.yasara.org). SymmDock was used to model the PEMV trimer [29].

### 2.2. Construction of CP Mutants

Sixteen CP mutants were created by site-directed mutagenesis (Figure 1, Table S1; QuickChange site-directed mutagenesis kit, Agilent Technologies, Santa Clara, CA, USA). The oligonucleotides were designed according to the QuickChange^®^ Primer Design Program (Agilent Technologies) and are listed in Table S2. Briefly, the pPER-1 plasmid containing the full-length wild-type PEMV1 [10] was digested with two unique site restriction enzymes *Spe* I and *EcoR* V (positions 3165 and 4717, respectively, of the full-length PEMV1 genome). The fragment of 1548 nucleotides encompassing the CP gene was purified, then ligated into pGEM-5Zf(+) vector according to the supplier protocol, resulting in a subclone named pGEM-PEMV-CP. Finally, each of the 16 mutated CP genes was religated into pPER-1. All mutant subclones and clones were sequenced using a specific primer (For-PEMV1_3922_: 5′-TGGTAGAGTGATCCCCCAGG-3′) at the Iowa State University DNA Facility.

### 2.3. Plasmid Digestion and In Vitro Transcription of Viral RNAs

Plasmids were linearized with *Pst* I restriction enzymes for pPER-1 and CP mutants and *Sma* I for pPER-2. Capped in vitro transcripts were synthesized in 3 h at 37 °C by using 2 µg of linearized plasmids and the mMESSAGE mMACHINE^®^ T7 Ultra kit (Ambion, Austin, TX, USA) according to the manufacturer’s directions. The transcripts were purified and assessed by electrophoresis and visualization by non-denaturing agarose gel electrophoresis (1%).

### 2.4. Plant Growth Conditions and Viral Inoculation

Seeds of *P. sativum* (organic pea Progress #9, Garden Harvest Supply, Berne, IN, USA) were sown individually in 5.0 × 5.5 × 7 cm pots with Sunshine LC-1 mix soil (Professional growing mix—73–83% Canadian sphagnum peat moss, perlite, dolomite lime, SHU 521) in a climate chamber (20 ± 2 °C, 12 h light at approximatively 60 µmol·m^−2^·s^−1^). Seven days after sowing, seedlings were mechanically inoculated with 5 µL of two equal amounts of either PEMV1 or CP mutant and PEMV2 in vitro transcripts (IVT) using 800 ng of each IVT. IVTs mixes were rubbed on the first leaves using an abrasive powder (Carborundum, Fisher Scientific Co., Pittsburgh, PA, USA). The inoculated and newly emerged leaves of each plant were sampled for measurement of viral accumulation. The positions of all inoculated plants for the experiment were randomized in the climate chamber. Control plants were mock-inoculated with 5 µL of RNA-free water.

### 2.5. Virus Detection in Pea Plants

#### 2.5.1. Quantification of Within-Host Accumulation by One-Step RT-PCR

Total RNA was extracted from the inoculated and youngest systemically infected leaves using TRIzol^®^ reagent (Ambion, Life Technologies, USA) followed by a normalized reverse transcription-quantitative PCR (RT-qPCR) method. Individual leaves, previously collected at 15 and 21 days post inoculation (dpi) and stored at −80 °C, were frozen in liquid nitrogen and then ground with zirconium oxide beads. Then, the leaf powder was treated with 8–10% (*v*/*w*) TRIzol^®^ reagent and chloroform. The nucleic acids were precipitated by adding 1 vol of isopropanol and 4 µL of linear polyacrylamide (5 mg/mL, Ambion) and resuspended in 30 µL of nuclease-free water. All RNA samples were then treated with 2U of TURBO DNase enzyme (TURBO DNA-free Kit; Ambion) to remove DNA contamination. DNase-treated total RNA was quantified with the QuBit^®^ fluorometer 2.0 (Invitrogen, Carlsbad, CA, USA) and the Quant-iT RNA HS Assay Kit according to the supplier’s procedure.

RNA quantification was performed in duplicate for each gene and sample by one-step real-time quantitative RT-PCR (qRT-PCR) in 384-well optical plates (CFX384 Touch™ Real-Time PCR Detection System, Bio-Rad, Hercules, CA, USA) using SYBR^®^ Green technology (iTaq One-Step RT-PCR Kit, BioRad). Each reaction was performed in 10 μL of 1× SYBR^®^ Green RT-PCR reaction mix, 0.125 µL of iScript Reverse Transcriptase, 300 nM each qPCR primer (Table S3), and 2 μL of either standards or diluted samples (30 ng/uL of total RNA). Cycling parameters were 50 °C for 10 min followed by reverse transcriptase inactivation for 5 min at 95 °C and then PCR cycling by 45 cycles of 10 s at 95 °C, 30 s at 62 °C. Fluorescence data were obtained via a melt-curve step from 65 °C to 95 °C with a gradual increase in temperature 0.5 °C/5 s and data acquired every 5 s. Normalized relative quantification (NRQ) for each sample was estimated using the standard curve and after transformation of the raw data using the Lin-RegPCR program [30,31] according to the Pfaffl formula [30]. A logarithmic transformation was applied to NRQ dataset.

#### 2.5.2. Sanger Sequencing

Viral RNA was checked by Sanger sequencing as follows. One microgram of total RNA extract positive for the virus by RT-qPCR, was reverse transcribed using Rev-CP_4755_ primer (5′-GATTCGGGTACGCCTTCATAG-3′) and SuperScript II RNase H—Reverse Transcriptase (Invitrogen, Life Technologies, Waltham, CA, USA), according to the manufacturer’s instructions. The CP ORF (ORF-3) was amplified with primers Rev-CP_4755_ and For-PEMV1_3922_ using Platinum^®^ Taq DNA Polymerase (Invitrogen) and 2 μL of 1:10 dilution cDNA. Cycling parameters were 94 °C for 2 min followed by 35 cycles of 30 s at 94 °C, 30 s at 60 °C, and 1 min at 72 °C, then 7 min at 72 °C. The PCR product was assessed by electrophoresis and visualization in a non-denaturing agarose gel (1%). The PCR products were sequenced using both forward and reverse PCR primers at the Iowa State University DNA Facility. CLC Main Workbench software package version 6.0 (CLC bio, Aarhus, Denmark) was used to analyze chromatograms for each sample.

#### 2.5.3. CP Detection by Western Blot

Thirty to 150 milligrams of infected leaf tissue collected from pea plants at 15 or 21 dpi was ground with liquid nitrogen, suspended in two volumes of 0.2 M sodium acetate buffer pH 6.0, then centrifuged at 12,000× *g* for 1 min. The total protein concentration of each sample (1:3 dilution) was estimated using Bradford reagent (Bio-Rad, USA) with bovine serum albumin (BSA) standards from 12.5 to 50 ng/μL. The samples were calibrated to the same amount of total proteins using 0.2 M sodium acetate buffer. The calibrated samples were mixed with 10× sample reducing agent and 4× lithium dodecyl sulfate buffer (LDS; Novex, Life Technology, Carlsbad, CA, USA) and boiled at 70 °C for 10 min. Fifteen microliters of the protein suspensions were loaded and separated in a 4–12% Bis-Tris Gel with 1× compound 2-(*N*-morpholino)ethanesulfonic acid (MES) buffer supplemented with an antioxidant (Novex). The same wild type (WT) positive control was loaded as reference in all 10 gels used for two independent experiments. Next, the proteins were transferred into an Amersham Protran Premium 0.45 NC membrane (GE Healthcare Life Sciences, Freiburg, Germany). The primary PEMV polyclonal rabbit antibody (AC Diagnostics, Inc., Fayetteville, AR, USA) as well as the secondary antibody goat HRP-conjugated anti-rabbit (Invitrogen, USA) used for western blots were diluted 1:2000. The chemiluminescence was detected with the Pierce ECL western blotting substrate (ThermoFisher Scientific, Rockford, lL, USA). GelQuant software was used to determine the density of bands at approximatively 22 kDa corresponding to CP. Five technical quantification replicates were done for each sample.

#### 2.5.4. Electron Microscopy

PEMV virions were partially purified from plants according Liu et al. [32]. Briefly, individual infected pea plants were harvested at 15 and 21 dpi. The plant material was ground in liquid nitrogen and resuspended in 1× *w*/*v* 0.2 M sodium acetate buffer (pH 6.0) and 1 volume of chloroform. The homogenized tissue suspension was clarified at 1200× *g* for 45 min (Sorval ST40R, ThermoFisher Scientific, Osterode am Harz, Germany), and the supernatant was mixed with 33.3% *v*/*v* of Triton X-100 then centrifuged again at 2000× g for 15 min. The virions were pelleted by ultracentrifugation at 78,000× *g* for 2 h at 4 °C and resuspended in 30 μL of 0.2 M sodium acetate buffer (pH 6.0). The suspensions were negatively stained with 2% uranyl acetate and examined using a JEOL 1200 transmission electron microscope (Iowa State University Microscopy and NanoImaging Facility). Negative control plants were mock-inoculated donor plants. Positive control plants were inoculated with wild-type viruses.

#### 2.5.5. Aphid Transmission Assays

The capacity for transmission by aphids was measured at 36–37 dpi, by allowing aphids to feed first on infected “donor” plants, and then on “recipient” plants. This time point was selected on the basis of previous experiments following within-host viral accumulation of PEMV1 WT in the non-inoculated upper leaves, which showed that viral density reached the maximum after approximately 21 days (unpublished data). We estimated the transmission efficiency of the wild-type and 16 CP mutant viruses using the pea aphid *Acyrthosiphon pisum* Harris [11,12]. This aphid colony *A. pisum* was reared on broad bean, *Vicia faba* in a growth chamber at 24 °C with a 12 h light/12 h dark cycle. 

A mixture of non-viruliferous nymphs and adults was sampled and starved for 2 h in glass tubes in groups of 40. These aphids were then placed on the top three leaves of each individual infected pea plant and kept in individual 4.5 × 14 cm insect rearing nylon bags during the time of acquisition. After a five-day acquisition feeding period, four or five aphids were placed on a 10–11-day-old healthy *P. sativum* recipient plant. We used one to three donor plants for each clone, and transferred aphids from each donor plant onto four to six recipient plants. After 72 h, approximatively 0.6 grams of systemic insecticide (Mantra^®^ 1G, Nufarm Americas Inc., Alsip, IL, USA) was added into each pot. Recipient plants were scored for PEMV1 infection 21 days after aphid infestation by evaluation of symptoms and viral detection by RT-PCR. Negative control plants were mock-inoculated donor plants.

### 2.6. Statistical Analysis

A contingency table chi-square test was used to compare each mutant to the wild-type treatment for their capacity to infect the inoculated and upper pea leaves. Data on viral accumulation by real-time RT-PCR were analyzed using ANOVA. Student’s *t*-test was used to investigate which mutants had a different accumulation level compared to the wild-type. The non-parametric Wilcoxon rank sum test was used for comparing the relative intensity of CP bands of WT to each mutant on the blots. A *p*-value of less than 0.05 was set as a statistically significant level throughout this study. Statistical analyses were carried out using JMP 12.0 (SAS Institute Inc., Cary, NC, USA) and a statistics server (http://www.socscistatistics.com/).

## 3. Results

### 3.1. CP Predicted Structure

In order to localize the domains on the surface of virions, we predicted the CP structure of PEMV1, by comparison with known structures of coat proteins of other viruses. The main templates used by I-TASSER to model the protein were *Ryegrass mottle virus* (RGMoV, *Sobemovirus* genus; PDB ID 2IZW), *Panicum mosaic virus* (PMV, *Tombusviridae* family; 4FY1), *Sesbania mosaic virus* (SeMV, *Sobemovirus* genus; 1SMV), Orsay virus-like particle (nematode virus; 4NWV), *Melon necrotic spot virus* (MNSV, *Tombusviridae* family; 2ZAH) and hepatitis A virus (HAV, *Picornaviridae* family; 4QPG). These have between 9% and 20% sequence identity of the whole template chains with the PEMV1 sequence. We report the predicted structures only for the first of five CP models proposed by the server (C-score −1.20, TM-score 0.56 ± 0.15, RMSD 7.8 ± 4.4 Å; Figure 1A). Although the protein topology TM score is <0.5, the estimated C-score is between −5 and 2, indicating that the protein structure has high accuracy. The local structure error prediction indicated that the first 45 aa belonging to the R-domain of the CP were less accurate, with primarily the first 19 residues having a very high modeling error [28]. By using the same approach, we verified the predicted structure of PLRV CP, obtained previously by Lee et al. [21]. The PLRV structure results were similar to those of Lee and collaborators, supporting the algorithm used in this study (Figure S1). The conserved structure of the S-domain among small, icosahedral RNA plant viruses was revealed with the eight β-strand barrel that forms the jelly-roll structure in the S-domain [33]. The surface epitopes on the loops are located between β strands H and I and between β strands B and C. The helix between β strands C and D and the loop between β strands G and H including the antigenic epitope 10, encompass the residues of the two acidic patch domains [20,25]. The helix between β strands E and F is also predicted to be on the surface of the virus particle, as described by [26] for the BWYV model. This region is another potential site for interaction between the virus and aphid vector. Finally, the N-terminal arginine-rich domain (residues 1–45; Figure 1A,B) is highly variable between PEMV1 and PLRV, i.e., the internal domain that interacts with the viral RNA where the 3D predictor scores are low (Figure S1). We verified the PEMV1 CP model by using Phyre2 server (http://www.sbg.bio.ic.ac.uk/phyre2), showing a similar predicted structure although the algorithms are different (i.e., homology detection methods, Figure S2).

### 3.2. PEMV Replication and Systemic Infection

Sixteen mutant viruses were generated based on the alignment of the CP sequences of PEMV, PLRV, and BWYV (Figure 1A). The sites to mutate were chosen after mapping the epitopes into close proximity at the middle of the trimer as well as regions predicted to be on the surface of the virion resulting from structure predictions. Some mutants were also designed based on knowledge of biological functions (i.e., virion assembly, transmission by aphids, and/or long-distance movement in the host plant) of certain aa in the CP of PLRV and BWYV [19,21,22]. Mutants N-R10/14-6R and N-R10/14-6K are in the R domain, which was shown to play a role in aphid transmission for PLRV (Kaplan et al., 2007). These two mutants were generated by deleting three aa at positions 11 to 13 from the original sequence (Figure 1A, Table S1). Mutant DQK76/81 was constructed by deletion of three aa between position 76 and 81 and mutants TTK120, LDT130, and DTW131 by insertion of two aa in the CP sequence, disrupting the structural motif of the protein (see Figure 1A, Table S1) in these domains, predicted on the surface of the virion at positions 120 to 122 and 129 to 132, respectively. The mutant K89A is located in the second acidic patch region, where alanine substitution of E109 reduced the efficiency of PLRV transmission [21]. Finally, mutants D149A, Q150A, P151A, W152A, Y153A, E154A, S155A/N156Ø, K157A, and D158A are all in an acidic patch region including epitope 10, where the YESNKDQ residues are located (Figure 1A,B).

We evaluated the infectivity rate of PEMV1 (WT and mutants) and PEMV2 independently in 250 co-inoculated leaves at 15 and 21 dpi. Using RT-qPCR, we detected 245 and 248 positive samples for PEMV1 and PEMV2, respectively (Table 1). Thus, PEMV2 replicated in three co-inoculated leaves in which PEMV1 did not replicate. In only two of the 250 inoculated leaves, neither virus accumulated. All 16 CP mutants accumulated in the inoculated pea leaf to the same level as the wild-type (WT versus mutants, chi-square test, *p* > 0.18) at 15 and 21 dpi. In the non-inoculated upper leaves, the infectivity rate was calculated as the number of samples positive for virus relative to the number of positive inoculated leaves. The infectivity rate of nine mutants was not significantly different to that of PEMV1 WT at 15 and 21 dpi (*p* ≥ 0.17; Table 1A). Except for DTW131, all of these mutants were in the acidic patch domain including the epitope 10 (yellow domain, Table 1A and Figure 1A). Five mutants, N-R10/14-6R, K89A, TTK120, LDT130, and W152A, had significantly higher infectivity rate of upper leaves compared to WT at 21 dpi (*p* ≤ 0.024). The mutant E154A was not detected in inoculated leaves at 15 dpi, while no samples were positive for Q150A at 21 dpi. Only K89A also showed significantly greater infectivity than the WT at 15 dpi (χ^2^ = 8.65, *p* = 0.00328, Table 1A). We conclude that all 16 CP mutants were able to move long-distance in pea plants, although the efficiency of long-distance movement was variable.

The long-distance movement of PEMV2 was more efficient than PEMV1: half of the non-inoculated upper leaves were positive for wild-type PEMV2 RNA (Table 1B). Moreover, some mutants could help the movement of PEMV2. For example, the number of samples containing PEMV2 in co-infection with D149A was significantly higher than with WT at 15 dpi (chi-square test, χ^2^ = 3.96, *p* = 0.047). Six other mutants, DQK76/81, K89A, TTK120, LDT130, P151A, and W152A, significantly improved PEMV2 infectivity compared to WT at 21 dpi (*p* ≥ 0.049; Table 1B).

**Figure 2:**
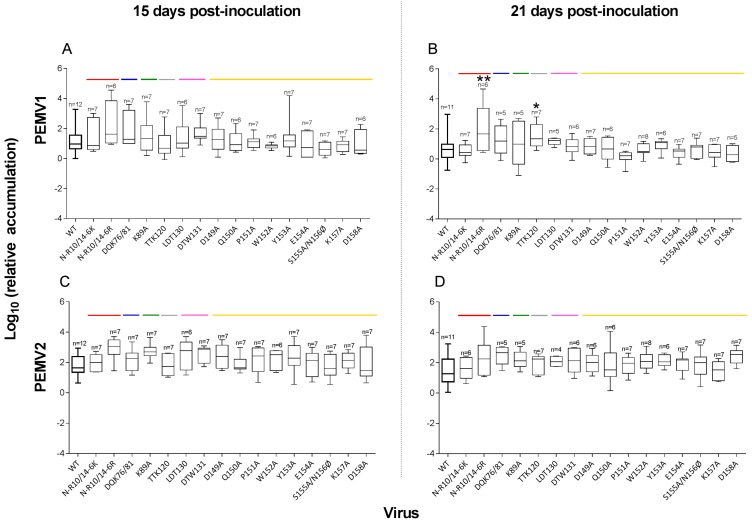
Accumulation of WT and mutant PEMV within inoculated leaves. (**A**,**B**) PEMV1; (**C**,**D**) PEMV2, WT and mutant accumulation at 15 and 21 days post-inoculation (dpi) of pea leaves, respectively. Virus accumulation was determined by RT-qPCR with reference to actin. All the mutants are able to replicate in the inoculated leaves as the same level as WT. The horizontal line within each box indicates the median value (50% quantile), while the box delimits the 25% and 75% quantiles. Colored lines correspond to the location of each mutant in specific coat protein domains as depicted in Figure 1. The number (*n*) of infected leaves tested for each virus are indicated with significant differences relative to WT shown (* *p* < 0.05; ** *p* < 0.01).

The level of accumulation of PEMV1 and 2 was estimated in the inoculated and non-inoculated upper leaves by real-time RT-PCR. In the inoculated leaves, we did not observe any significant difference in replication among the PEMV1 mutants at 15 dpi (ANOVA on log_10_ (accumulation related to actin), d*f* = 16, *F* = 1.37, *p* = 0.17, Table 2 and Figure 2A) ranging from 0.62 ± 0.41 (S155A/N156Ø) to 2.24 ± 1.49 (R10/14-6R). However, a significant difference in accumulation between viral clones was observed at 21 dpi (d*f* = 16, *F* = 2.25, *p* = 0.0082, Table 2 and Figure 2B). Mutants N-R10/14-6R and TTK120 accumulated to significantly higher levels than PEMV1 WT (Student’s *t*-test, *p* ≤ 0.0397). We did not observe significant differences in the accumulation of PEMV2 in the presence of any of the mutant PEMV1 RNAs (ANOVA, d*f* = 16, *p* ≥ 0.13, Table 2 and Figure 2C,D). The results did not differ when β-tubulin mRNA was used as the reference (Table 2, Figure S3).

**Figure 3:**
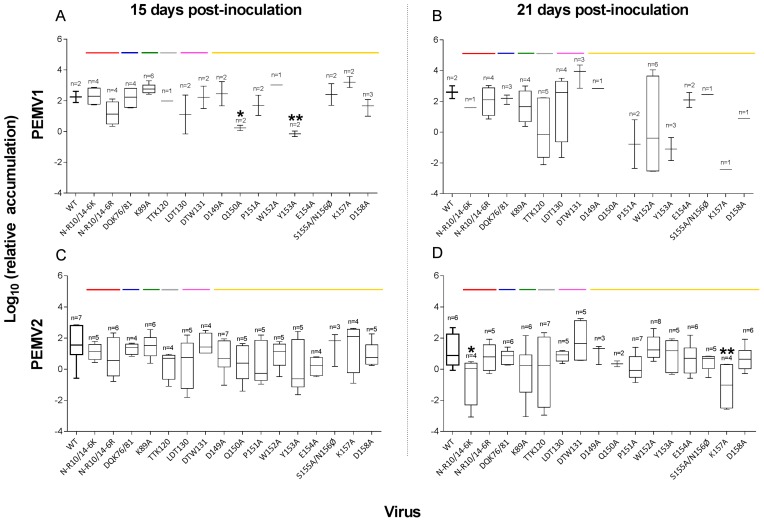
Accumulation of wild-type and mutant PEMV within upper leaves of inoculated pea plants. (A,B) PEMV1 and (C,D) PEMV2 RNA accumulation in upper leaves at 15 and 21 dpi of lower leaves. Viral RNA was quantified with real-time RT-PCR using actin as a reference. Colored lines correspond to the location of each mutant in the coat protein as shown in Figure 1A. The numbers of infected leaves test for each virus are shown with significant differences relative to WT indicated (* *p* < 0.05 and ** *p* < 0.01).

In the non-inoculated upper leaves of inoculated plants, there was a significant difference in accumulation between PEMV1 WT and some mutants at 15 dpi (ANOVA, d*f* = 13, *F* = 4.15, *p* = 0.0011, Table 2 and Figure 3A). Q150A and Y153A showed significantly lower accumulation than WT (Student’s *t*-test, *p* ≤ 0.010; Figure 3A) with two replicates per mutant. No difference in viral accumulation was observed at 21 dpi for those mutants that could be compared (ANOVA, d*f* = 10, *F* = 1.33, *p* = 0.26). However, because some mutants appeared in only one leaf, they were excluded from variance analysis. Thus, the significance could not be calculated, although they may well be less efficient in replication or movement than WT or the other mutants.

To determine if some mutants reverted to WT or acquired second-site mutations in the CP, the full CP gene of progeny virus in the systemic non-inoculated leaves was sequenced after amplification by reverse transcription and PCR. Two samples of viral progeny were analyzed for each mutant (except for DTW131, where only one was sequenced). We detected no reversions or additional mutations in the CP sequence of the progeny of 14 mutants, nor did we detect any changes in the WT progeny. However, an Asp_116_ to Gly mutation was observed in one plant infected by mutant K157A. None of the samples infected by P151A were positive by PCR. Hence, there was no progeny of this mutant virus to sequence. 

Accumulation of PEMV2 in the upper leaves was not affected by co-infection of PEMV1 mutants compared to WT at 15 dpi (ANOVA, *p* = 0.42; Table 2 and Figure 3C). However, at 21 dpi, PEMV2 accumulated to a lower level in upper leaves after co-inoculation with N-R10/14-6K and K167A (ANOVA, *n* = 4, *p* ≤ 0.019, Figure 3D). In plants co-inoculated with either of these mutants and PEMV2, only one replicate of each showed presence of the mutant PEMV1 in the upper leaf (compare Figure 3B,D). In three replicates of each of these infections, PEMV2 alone was detected in the upper leaf. Thus, PEMV2 sometimes moves to upper leaves in the absence of PEMV1. The results did not differ using the data set with β-tubulin mRNA as the reference except for one mutant K89A, which were significant at 21 dpi using tubulin gene (Student’s test, *p* = 0.041; Figure S4) while non-significant with actin gene (*p* = 0.054).

**Figure 4:**
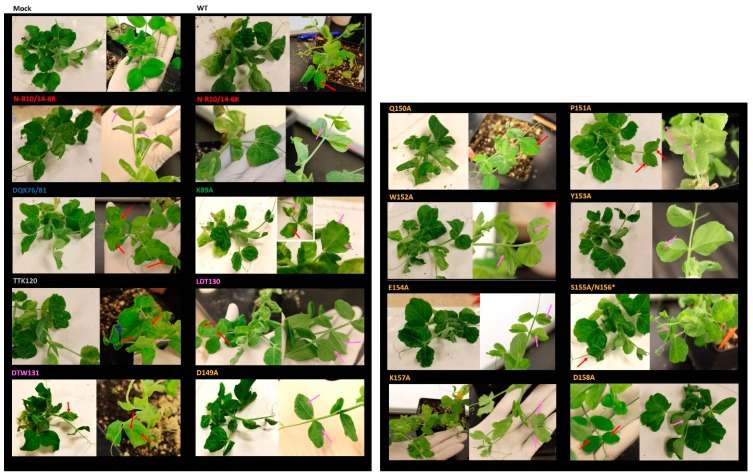
Symptoms of pea plants infected with WT and mutant PEMV. Red arrows indicate the position of mild chlorotic vein flecking on the topside and the pink arrows indicate vein enations on the underside of the leaves. The plants were randomly photographed between 21 and 30 dpi. Two independent plants for each clone are shown.

The mutants in the R-domain (N-R10/14-6K and N-R10/14-6R) as well as K89A showed slightly more severe symptoms, appearing earlier than WT in the upper leaves (i.e., 4–5 dpi for mutants versus 6–7 dpi for WT). Unlike plants infected with wild-type virus, plants infected with mutants LDT130, DTW131 and P151A did not show any symptoms during the first weeks, although some enations appeared after 21 dpi under the youngest leaves. Finally, K157A and D158A developed only mild symptoms of chlorosis and chlorotic vein flecking compared to WT during all times of infection (Figure 4).

### 3.3. CP Expression and Virion Assembly

To determine the effects of CP mutations on CP accumulation, we performed western blots with anti-PEMV virion antibody. Some symptomatic plants positive for virus by real-time RT-PCR were harvested to evaluate the quantity of coat protein by western blot. We increased the number of samples for CP detection by Western blot by harvesting all “donor” pea plants used to feed aphids (positive by real-time RT-PCR) from the transmission experiment described below.

**Figure 5:**
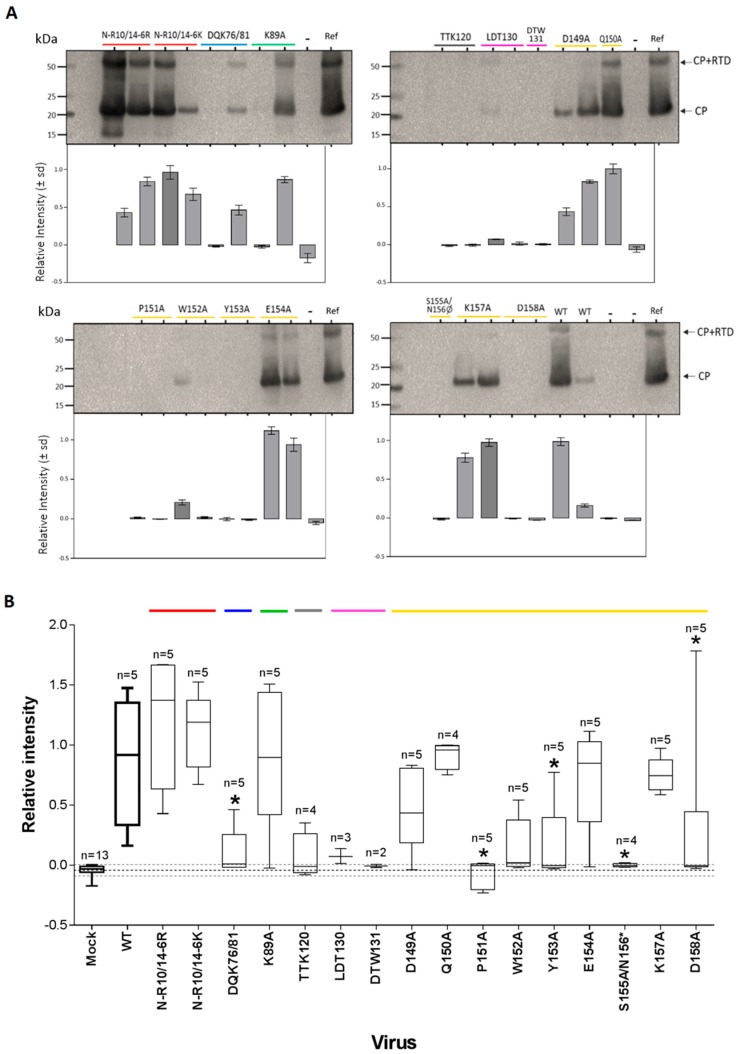
Detection of virion structural proteins in pea plants infected with WT or CP mutants by Western blot. (**A**) Western blot images (upper) were scanned and processed using GelQuant to estimate the relative amount of CP (histogram below) to an external positive control (named Ref); (**B**) relative intensity of CP bands pooling biological replicates from two independent experiments. The quantity of CP in the upper leaf for each virus is shown, with significant differences relative to WT indicated (* *p* < 0.05). Mutations in the S domain (grey domain) resulted in a weak expression of structural proteins of certain mutants previously positive by RT-qPCR. Only the mutant Q150A was efficiently transmitted by aphids. Leaves from plants infected with WT PEMV and uninfected leaves were used as positive and negative controls, respectively. Colored lines indicate the region in the CP for each mutation, as indicated in Figure 1A.

The major coat protein of approximately 22 kDa was not detected by western blot in every sample (Figure 5, Figure S5) although viral RNAs were always detected by RT-qPCR. In order to increase the number of biological replicates for statistical analysis, we pooled the data from two independent experiments (i.e., experiments of viral accumulation and transmission). The intensity of bands was significantly different between viruses (non-parametric test, χ^2^ = 42.3, d*f* = 16, *p* = 0.0004; Figure 5B). We classified our mutants into four different groups according to CP band intensity relative to WT and the number of positive replicates. First, the mutants N-R10/14-6R, N-R10/14-6K, Q150A, and K157A gave intense bands on blots for all replicates and in both experiments (WT versus mutant, Wilcoxon rank sum test, *p* ≥ 0.39; Figure 5, Figure S5). Second, CP bands for the mutants K89A, D149A, and E154A were not significantly different to those of WT (*p* ≥ 0.18; Figure 5B). Some replicates had a lower relative intensity or no CP detection. Third, the mutants DQK76/81, TTK120, LDT130, W152A, Y153A, and D158A had only one or two samples that were positive with low-level detection of CP (relative intensity range values of 0.00–0.01; Figure 5, Figure S5). These values are only slightly higher than the range of values for the mock (mean ± sd, −0.0425 ± 0.047) and hence it is difficult to conclude that CP was present. Finally, CP detection for the mutants DTW131, P151A, and S155A/N156Ø was not significantly different to the mock (*p* ≥ 0.05; Figure 5B).

**Figure 6:**
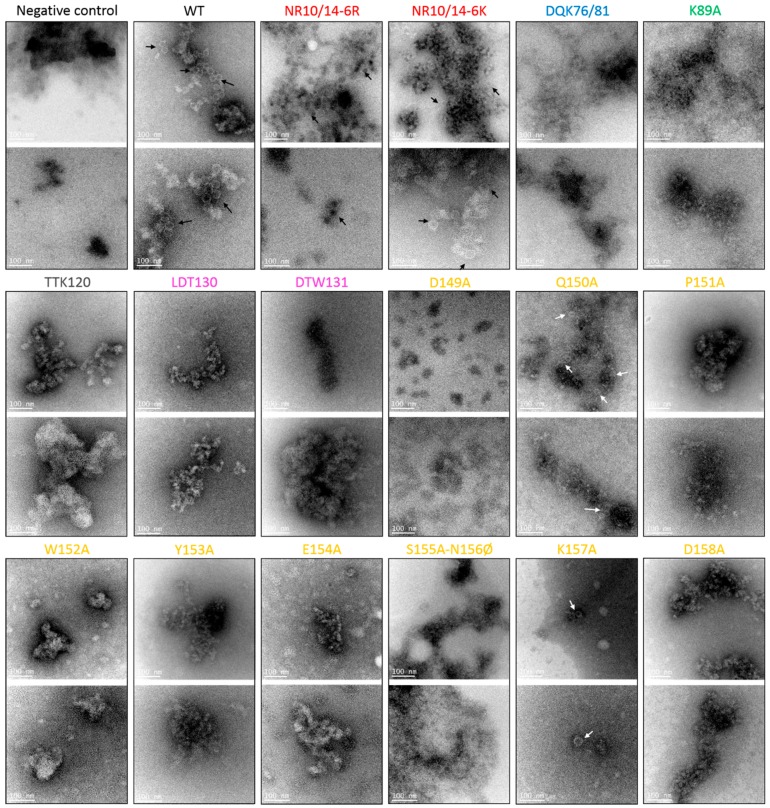
Detection of virions from pea plants infected with WT or CP mutants of PEMV. Crude virion extracts were negatively stained with uranyl acetate and visualized by transmission electron microscopy. Arrows indicate the position of virions. They were detected for only four of the 16 mutant viruses, NR10/14-6R, NR10/14-6K, Q150A, and K157A. Images are representative of four to 12 images examined for each sample. Scale bars, 100 nm.

A crude virus preparation was performed by ultracentrifugation of extracts from one or two randomly selected infected plants used for the western blots from the experiment of viral accumulation. The resulting preparation was examined by transmission electron microscopy. In addition, we included three positive samples infected by the WT and a mock-infected plant to validate the method used. The samples were analyzed by Iowa State University Microscopy and NanoImaging Facility. Only four mutants, N-R10/14-6R, N-R10/14-6K, Q150A, and K157A, yielded virions similar to the WT, while no virions were detected for any of the 12 other mutants (Figure 6). Interestingly, no virions were detected for samples infected by the mutants K89A and E154A, although high CP expression was detected by western blot (Figure 5).

### 3.4. Aphid Transmission

We evaluated the capacity of each CP mutant for transmission by the pea aphid, *A. pisum*. Aphids were fed for five days on plants shown to be infected by the presence of symptoms and by RT-PCR. Aphids were then transferred to healthy pea plants for three days. A total of 51 donor plants were used at the same time for this experiment. Three donor plants were the source of virus for each of 13 mutants, while two infected plants were used for TTK120, LDT130, and WT. Only one positive donor plant was used for the mutant DTW130. We included five healthy pea plants in the experiment as negative controls. None of the 24 recipient plants from the negative controls were positive for virus. This validated that no cross-contamination was detected during the process of aphid transmission. The WT was highly transmissible under these experimental conditions; the two biological replicates had a transmission rate of 7/9 and 14/16. Of all the mutants, only Q150A was highly transmissible for two of the three biological replicates (ratio positive plants/total of recipient plants tested equal to 4/6 and 5/6 plants). The progeny of the third replicate was not transmissible in this experiment (0/6 positive plants for Q150A). None of the other mutants that yielded virions observable by TEM (N-R10/14-6R, N-R10/14-6K, and K157A, Figure 6) were transmissible by aphids. Also, the other mutants that produced RNAs and CP detected at the same intensity (by western blot; Figure S5) as the WT were not transmissible by aphids.

## 4. Discussion

### 4.1. Replication in Inoculated Leaves

All 16 of the CP mutants were able to initiate replication and accumulate in the inoculated tissues, at levels similar to the WT. This is consistent with previous studies of two poleroviruses, which detected replication of all CP mutants of both BWYV transfected into protoplasts [19] and PLRV in agroinfiltrated plant tissues [21,22]. Moreover, replication of PEMV2 was not significantly different among plants co-infected with the WT PEMV1 and the 16 CP mutants. Demler and co-workers demonstrated that PEMV2 replicates autonomously in plant tissues [8]. We can likely exclude technical problems during the mechanical co-inoculation of PEMV1 and PEMV2 into plants to explain the difference of RNA amount observed between PEMV1 WT and the mutants of PEMV1 in the inoculated leaves. 

Interestingly, N-R10/14-6R and TTK120 showed significantly higher accumulation at 21 dpi than the wild-type for reasons that are not clear (Figure 2). The mutant N-R10/14-6K differs from N-R10/14-6R by only the residue in position 6 in the R-domain. The WT clone used in our study has a lysine at this position while the N-R10/14-6R contains an arginine, which is also in this position in the original clone sequenced [7]. Clearly, either basic amino acid is sufficient at position 6, as N-R10/14-6K does not behave differently from WT in the inoculated leaves. As previously suggested [22], the basic R-domain presumably interacts with the viral RNA into the virion interior. Its role in PEMV replication in plant cells remains unknown.

### 4.2. Abilities of Mutants to Move in Plants

The evaluation of virus accumulation in the upper leaves of plants infected with CP mutants revealed unexpected results that contrast with previous studies of poleroviruses [19,21,22,26]. All mutants are able to move long-distance and replicate systemically in pea tissues, as detected by RT-qPCR, and by visualization of symptoms induced by infection. However, two mutants in epitope 10 (Q150A and Y153A) accumulated to lower levels than WT at 15 dpi (Figure 3). While some mutants replicate similarly to WT, we interpret these results cautiously because numerous mutants infected upper leaves of only one or two replicate(s). Moreover, the evaluation of the infectivity rate showed that the number of positive plants infected systematically differed among mutants at both 15 and 21 dpi. While almost all plants were infected by PEMV1 and PEMV2 in the inoculated leaves, the mutations in the CP clearly affect the long-distance movement of PEMV1, and in some cases PEMV2 (Table 2). Interestingly, the mutant K89A has a higher infectivity rate compared to WT at both sampling times. Moreover, this mutant also helps the movement of PEMV2 at 21 dpi (Table 2), and the symptoms are more severe and occur earlier after inoculation than they do in WT-infected plants (observed in three independent experiments; Figure 4). 

The passage from the inoculated to the upper leaves, crucial for the infection of the whole plant by PEMV, seems to be led by ORF-3 encoding the CP, the CP itself, or both by specific RNA–protein interactions. With this unique symbiotic model, PEMV1 is unable to move cell-to-cell or systemically via the phloem without PEMV2, which encodes the movement protein [10,13]. Skaf and collaborators [34] observed similar results on PEMV by studying the long-distance movement of deletion mutants in the CP. In fact, PEMV1 has the capacity to move long-distance via the phloem without being encapsidated by using a replication complex or double-membrane-bound vesicles associated with its genome [14]. This association forms a ribonucleoprotein complex protecting viral RNAs against ribonucleases in plants [35,36]. This differs from the polerovirus *Turnip yellows virus* (formerly called BWYV), in which assembly of virions is required for long-distance movement [37]. However, because PEMV2, like all umbraviruses, does not encode a coat protein, it must exploit alternative mechanisms for RNA protection and systemic spread in plants, in the absence of PEMV1. Umbravirus ORF-3 protein has been shown to stabilize viral RNA and facilitate long-distance movement through the phloem [38,39,40].

### 4.3. Capacity of Assembly and Transmission by Aphids

While a relatively similar amount of viral RNA was detected in new leaves for all mutants, the amount of CP and CP-RTD varied greatly (Figure 5, Figure S5). Mutants P151A and S155A/N156Ø expressed no CP. We do not know whether the reduced accumulation of CP by certain mutants is due to a defect in its synthesis or instability of the protein, or both. For several positive-sense RNA viruses, the amount of CP modulates the initiation of infectious cycle, translation, and/or replication in infected tissues (e.g., *Brome mosaic virus* [41]; *Potato virus* A [42]). Depending on the amount of CP, the virus can regulate the progression of virus infection from genome replication and translation to virion assembly. 

Although 14 of the 16 mutants accumulated CP, virions were observed by transmission electron microscopy in only four mutants (N-R10/14-6R, N-R10/14-6K, Q150A, and K157A; Figure 6). Interestingly, Q150A is the only mutant still transmissible by aphids. In fact, these results were not predictable by using only the measurement of genomic RNA accumulation by RT-qPCR and/or CP detection by Western blot. For example, Q150A replicated significantly lower than WT at 15 dpi with a low infectivity rate while E154A and K89A mutants replicated with the same amount of RNA and coat protein as WT, but no virions were observed. These results suggest that the virions of certain mutants could be unstable, leading them to not being purified properly by the protocol used in this study. However, we observed that the 12 mutants affected in virion assembly, including E154A and K89A (Figure 1B), are also not transmissible by aphids. Thus, although the formation of filamentous ribonucleoprotein particles protect viral RNAs from the plant’s defensive RNA-silencing response and ribonucleases during the virus spread in the plant [9], viruses are not transmissible with this alternative form and require stable and functional virions for that. 

Although the two mutations in the N-terminal R domain did not affect the assembly, none of the progeny containing these mutations was transmissible by *A*. *pisum*. This basic domain is predicted to interact with RNA in the interior of the virion, so its role in aphid transmission is unexpected. As we know, the arginine-rich RNA-binding motif can bind RNA with high affinity and recognize features of the RNA. This motif is found in other plant viruses, such as the *Bromoviridae* family, the *Sobemovirus* and *Tombusvirus* genera, and in human immunodeficiency virus Tat and Rev proteins [43,44,45]. The α-helical conformation of the motif requiring few specific aa is essential for RNA recognition, transactivation [45,46], and packaging [43]. Several authors observed similar effects of mutations in the R domain of PLRV CP, but transmission by aphids was not totally abolished [22,47]. Crystal structures of tomato bushy stunt virus, [33], and virions belonging to the *Sobemovirus* genus (e.g., RGMoV [48]; SeMV [49]), which are closely related to *Enamovirus*, reveal that viral RNA interacts with the flexibly-linked arms of the R-domain, which can be disordered in A/B subunits of the trimer. The aa modifications in the R-domain (mutants N-R10/14-6R and N-R10/14-6K) potentially change its interaction, conformation, and/or exposure outside the virions, as shown in *Cowpea chlorotic mottle virus* [43]. This may disrupt the interaction of the PEMV virions with the receptor(s) in the gut, such as alanyl aminopeptidase N [6]. Alternatively, the deletions and/or mutation in the R-domain may preclude or weaken binding of CP to viral RNA. Thus, the virions may not contain RNA or free RNA cannot bind CP and is thus potentially unable to initiate a new infection. 

Although none of the mutations in the domains of the “acidic patch” (green and yellow domains on Figure 1A) affect the systemic movement of PEMV, many affected virion assembly or stability (Figure 1 and Figure 6). By superimposing the predicted PEMV and PLRV CP structures, we observed that Pro_151_ of PEMV CP is situated near the trimer center and can interact with the neighboring residues of the “acidic patch.” This amino acid appears to be necessary for the formation of the trimer (Figure 1D–F, Figure S1). Moreover, we noticed that the mutant P151A did not produce any CP detected by western blot and its mild symptoms appeared late after inoculation compared to the WT (Figure 4). Nor were we able to amplify the CP gene of P151A progeny. Interestingly, the two polerovirus models located a conserved tryptophan in the middle of the trimer interface to stabilize virion formation (Trp_166_ and Trp_171_ for BWYV and PLRV, respectively [19,21]). PEMV1 has a tryptophan in position 152 but our predicted model does not locate its residues in the center of the trimer. However, it may play a role, as no assembly is observed in the W152A mutant (Figure 6). Mutation of the aa residues near the center of the trimer did not affect the assembly of PLRV, but did affect the transmission [21]. We did not observe the same pattern for PEMV. Gln_150_ is adjacent to Pro_151_, but is predicted to be exposed inside the loop G-H (Figure 1A). The Q150A mutation did not affect assembly or transmission relative to the WT. Lys_157_ is also predicted to be oriented inside loop G-H. K157A also did not affect assembly of the virions, but it eliminated aphid transmissibility. Lee and co-workers (2005) observed the same results by altering Glu_156_ in PLRV CP (Figure 1B). This amino acid is predicted to be adjacent to the threefold axis of symmetry in PLRV virion.

The mutants LDT130 and DTW131 are predicted in a domain in contact between CP monomers within β-strand F in close proximity to the threefold axis. These two mutants overlap with the position of Lys_150_ in the PLRV 3D model, which was described as a “hot spot” for virus–host protein interaction [24]. In PLRV, all mutants within or bordering β-strands did not assemble [22]. Our results with mutants LDT130 and DTW131 are consistent with this model. Although PEMV1 does not have a lysine as “hot spot” as in PLRV and BWYV (positions 150 and 144, respectively; Figure 1B), our model structure identified Trp_132_, which is exposed on the surface of the virion, as represented in Figure 1D. This amino acid could subsequently be evaluated in further studies to evaluate the potential CP–host and CP–aphid interactions. 

The CP–CP interactions at the β-annulus are also necessary to form the virion, as schematized in Figure 1C, although not studied here. By aligning PEMV CP to PLRV and BWYV models, we mapped the epitope GTGGTDVAGHY at aa 166–176 of PEMV1 (Figure 1B) based on the peptide identified in the C-terminal region of PLRV CP [23]. This epitope is potentially involved in different protein interactions, as described in the PLRV model [23]. GNG deletion prevents virion assembly and disrupted the close interactions between CP monomers with Lys_188_ in PLRV CP [22,23]. Although no lysine is available in this epitope, our predicted PEMV model identified Thr_167_ as the amino acid allowing the interaction between CP monomers at the β-annulus (Figure 1G,H). This epitope in the C-terminal of CP may interact with the N-terminal of RTD protein when incorporated into the virion. This minor protein was described as involved in luteovirus transmission [50,51,52,53,54,55]. Although the readthrough domain of PEMV (33 kDa protein) is not essential for virus stability into the vector, it can indeed affect the transmission [32]. All the CP mutants in this study were able to express as a translational readthrough (RT) fusion with the 21 kDa CP detectable by western blot [16]. In summary, we have characterized mutants that provide guidance for future research on interactions involved in aphid transmission and virus assembly and movement, and for interpretation of high-resolution structures to be determined by X-ray crystallography or cryoelectron microscopy.

## Figures and Tables

**Table 1:** Infectivity of wild-type (WT) and mutant *Pea enation mosaic virus* (PEMV). PEMV 1 (**A**) or PEMV2 (**B**) was detected using real time PCR for wild-type and mutant viruses at 15 and 21 days post-inoculation (dpi). The number of positives over the total number of plants tested is shown. While virus was detected in almost all of the inoculated leaves, significant differences were noted for detection of virus in upper leaves.

**(A) PEMV-1 Infectivity**									
	**Location in CP**	**Inoculated Leaves**		**Upper Leaves ^1^**					
		**15 dpi**	**21 dpi**	**15 dpi**		**21 dpi**		**15 + 21 dpi**	
**Wild-Type**	**-**	**12/12**	**11/12**	**2/12**		**2/12**		**4/24**	
N-R10/14-6K	R-domain	7/7	7/7	4/7	#	1/7		5/14	
N-R10/14-6R	R-domain	6/6	6/6	4/7	#	4/6	*	8/13	*
DQK76/81	Helix αC-D	7/7	5/5	4/7	#	3/5		7/12	*
K89A	Loop C-D	7/7	5/5	6/7	*	4/5	*	10/12	***
TTK120	Helix αE-F	7/7	7/7	1/7		5/7	*	6/14	
LDT130	Barrel βF	6/7	5/5	2/6		5/5	**	7/11	*
DTW131	Barrel βF	7/7	6/6	2/7		3/6		5/13	
D149A	Loop G-H	7/7	6/7	2/7		1/6		3/13	
Q150A	Loop G-H	6/6	6/6	2/6		0/6		2/12	
P151A	Loop G-H	7/7	7/7	2/7		2/7		4/14	
W152A	Loop G-H	6/6	8/8	1/6		6/8	*	7/14	*
Y153A	Epitope 10	7/7	6/6	2/7		3/6		5/13	
E154A	Epitope 10	7/7	7/7	0/7		2/7		2/14	
S155A/N156Ø	Epitope 10	7/7	7/7	2/7		1/7		3/14	
K157A	Epitope 10	7/7	7/7	2/7		1/7		3/14	
D158A	Epitope 10	6/7	6/7	3/6		1/6		4/12	
**(B) PEMV-2 Infectivity**									
	**Location in CP**	**Inoculated Leaves**		**Upper Leaves ^1^**					
**Co-Inoculated with:**		**15 dpi**	**21 dpi**	**15 dpi**		**21 dpi**		**15 + 21 dpi**	
**PEMV-1 Wild-Type**	**-**	**12/12**	**11/12**	**7/12**		**6/12**		**13/24**	
N-R10/14-6K	R-domain	7/7	7/7	5/7		4/7		9/14	
N-R10/14-6R	R-domain	6/6	6/6	6/6	#	5/7		11/13	#
DQK76/81	Helix αC-D	7/7	5/5	4/7		6/6	*	10/13	
K89A	Loop C-D	7/7	5/5	6/7		6/6	*	12/13	*
TTK120	Helix αE-F	7/7	7/7	4/7		7/7	*	11/14	
LDT130	Barrel βF	6/7	5/5	5/6		5/5	*	10/11	*
DTW131	Barrel βF	7/7	6/6	4/7		5/6		9/13	
D149A	Loop G-H	7/7	7/7	7/7	*	3/7		10/14	
Q150A	Loop G-H	6/6	6/6	5/6		2/6		7/12	
P151A	Loop G-H	7/7	7/7	5/7		7/7	*	12/14	*
W152A	Loop G-H	6/6	8/8	5/6		8/8	*	13/14	*
Y153A	Epitope 10	7/7	6/6	5/7		5/6		10/13	
E154A	Epitope 10	7/7	7/7	4/7		6/7		10/14	
S155A/N156Ø	Epitope 10	7/7	7/7	3/7		5/7		8/14	
K157A	Epitope 10	7/7	7/7	4/7		4/7		8/14	
D158A	Epitope 10	7/7	7/7	5/7		6/7		11/14	

^1^ Significant effects # 0.05 < *p* < 0.075, * *p* < 0.05, ** *p* < 0.005, and *** *p* < 0.0005 (Student’s *t*-test).

**Table 2:** Effects of PEMV coat protein mutations on PEMV1 and PEMV2 accumulation relative to two housekeeping gene RNAs at 15 and 21 dpi.

Quantitative Trait	Leaf	Factor	15 dpi			21 dpi		
			d*f*	*F*	*p* ^1^	d*f*	*F*	*p* ^1^
PEMV1 Viral Accumulation relative to actin	Inoculated	Clones	16	1.3746	0.1694	16	2.2478	0.0082 *
	upper		13	4.1487	0.0011 **	10	1.3291	0.2652
PEMV2 Viral Accumulation relative to actin	Inoculated	Clones	16	1.4679	0.1258	16	0.9635	0.5021
	upper		16	1.0486	0.4203	16	1.9346	0.0303 *
PEMV1 Viral Accumulation relative to β-tubulin	Inoculated	Clones	16	1.1967	0.2838	16	2.1897	0.0109 *
	upper		13	3.1809	0.0063 *	10	1.5485	0.1764
PEMV2 Viral Accumulation relative to β-tubulin	Inoculated	Clones	16	1.3484	0.1830	16	0.8879	0.5848
	upper		16	0.8297	0.6483	16	2.1571	0.0141 *

^1^ Significant effects * *p* < 0.05 and ** *p* < 0.005 (ANOVA).

## Supplementary Materials

The following are available online at www.mdpi.com/1999-4915/8/11/312/s1, Figure S1: Predicted 3D structure of PLRV coat protein (CP) and comparison to PEMV CP; Figure S2: Overlay of predicted 3D structures of PEMV1 coat protein (CP) using Phyre2 and I-TASSER servers; Figure S3: Accumulation of wild-type and mutant PEMV within inoculated leaves relative to β-tubulin reference; Figure S4: Accumulation of wild-type and mutant PEMV within upper leaves of inoculated pea plants relative to β-tubulin reference; Figure S5: Detection of virion structural proteins following aphid inoculation of pea plants with wild-type or CP mutants by western blot; Table S1: Specific amino acid changes made for each of the 16 mutations made in PEMV CP; Table S2: Sequences of primers used for generation of mutant PEMV-1; Table S3: Sequences of primers used for quantification of PEMV1 and 2, and of transcripts for two housekeeping genes.
