# Supplementary material for: Role of Pea Enation Mosaic Virus Coat Protein in the Host Plant and Aphid Vector
Source: Viruses. 2016 Nov 18;8(11):312. doi: 10.3390/v8110312 (PMC5127026; doi:10.3390/v8110312)
Supplement: Supplementary file 1 [file viruses-08-00312-s001.docx]

Supplementary Materials: Role of Pea Enation Mosaic Virus Coat Protein in the Host Plant and Aphid Vector

Juliette Doumayrou, Melissa Sheber, Bryony C. Bonning, and W. Allen Miller

**Table S1.** Specific amino acid changes made for each of the 16 mutations in *Pea enation mosaic virus* (PEMV) coat protein (CP).

| **Mutant Name** | **Current Seq.** | **AA Change** | **Mutated Sequence** | **Genome Changes ^1^** |
| --- | --- | --- | --- | --- |
| N-R10/14-6R | KSKANQRR | 6…10-14 | **R**SKA**N--R** | M and D |
| N-R10/14-6K | KSKANQRR | 10-14 | N--R | D |
| DQK76/81 | DCQCIK | 76-81 | D-Q--K | D |
| K89A | QKY | K 89A | QaY | M |
| TTK120 | TTK | 120 | TtTkK | I |
| LDT130 | LDT | 130 | LlDtT | I |
| DTW131 | DTW | 131 | DdTwW | I |
| D149A | GDQ | D 149 A | GaQ | M |
| Q150A | DQP | Q 150 A | DaP | M |
| P151A | QPW | P 151 A | QaW | M |
| W152A | PWY | W 152 A | PaY | M |
| Y153A | WYE | Y 153 A | WaE | M |
| E154A | YES | E 154 A | YaS | M |
| S155A/N156Ø | ESNK | 155-156 | EaK | M and D |
| K157A | NKD | K 167 A | NaD | M |
| D158A | KDQ | D 168 A | KaQ | M |

^1^ M: mutation; D: deletion and I: insertion of amino acid(s).

**Table S2.** Sequences of primers used for generation of mutant PEMV-1.

| **Named** | **Orientation Primer ^1^** | **Primer Sequence ^2^** |
| --- | --- | --- |
| N-R10/14-6K | For | 5′-gactagatcgaaatcaaaagctaataggagaaggccta-3 |
|  | Rev | 5′-taggccttctcctattagcttttgatttcgatctagtc-3′ |
| N-R10/14-6R | For | 5′-gactagatcgagatcaaaagctaataggagaaggccta-3 |
|  | Rev | 5′-taggccttctcctattagcttttgatctcgatctagtc-3′ |
| DQK76/81 | For | 5′-ggaccctcctccgaccagaagggaaacctagc-3′ |
|  | Rev | 5′-gctaggtttcccttctggtcggaggagggtcc-3′ |
| K89A | For | 5′-ggaaacctagccgcttaccaagcatataggatcgtatggttaaa-3′ |
|  | Rev | 5′-tttaaccatacgatcctatatgcttggtaagcggctaggtttcc-3′ |
| TTK120 | For | 5′-gacacctccacaactactaagaagaaggccgccga-3′ |
|  | Rev | 5′-tcggcggccttcttcttagtagttgtggaggtgtc-3′ |
| LDT130 | For | 5′-cgccgatgtagtgttgcttcttgacactacttggaacattc-3′ |
|  | Rev | 5′-gaatgttccaagtagtgtcaagaagcaacactacatcggcg-3′ |
| DTW131 | For | 5′-gatgtagtgttgcttgacgacacgtggtggaacattcgttctaatgg-3′ |
|  | Rev | 5′-ccattagaacgaatgttccaccacgtgtcgtcaagcaacactacatc-3′ |
| D149A | For | 5′-tcgtgaaattcttggtgcacaaccgtggtacgagt-3′ |
|  | Rev | 5′-actcgtaccacgttgtgcaccaagaatttcacga-3′ |
| Q150A | For | 5′-tttcggtcgtgaaattcttggtgatgcaccgtggtacgag-3′ |
|  | Rev | 5′-ctcgtaccacggtgcatcaccaagaatttcacgaccgaaa-3′ |
| P151A | For | 5′-tgaaattcttggtgatcaagcatggtacgagtcc-3′ |
|  | Rev | 5′-ggactcgtaccatgcttgatcaccaagaatttcac-3′ |

**Table S2.** *Cont.*

| **Named** | **Orientation Primer ^1^** | **Primer Sequence ^2^** |
| --- | --- | --- |
| W152A | For | 5′-aaattcttggtgatcaaccggcatacgagtccaataaggatc-3′ |
|  | Rev | 5′-gatccttattggactcgtatgccggttgatcaccaagaattt-3′ |
| Y153A | For | 5′-tcttggtgatcaaccgtgggcagagtccaataaggatcag-3′ |
|  | Rev | 5′-ctgatccttattggactctgcccacggttgatcaccaaga-3′ |
| E154A | For | 5′-gatcaaccgtggtacgcatccaataaggatcagt-3′ |
|  | Rev | 5′-actgatccttattggatgcgtaccacggttgatc-3′ |
| S155A/N156Ø | For | 5′-atcaaccgtggtacgaggcaaaggatcagttctttttc-3′ |
|  | Rev | 5′-gaaaaaagaactgatcctttgcctcgtaccacggttgat-3′ |
| K157A | For | 5′-caaccgtggtacgagtccaatgcagatcagttctttttcct-3′ |
|  | Rev | 5′-aggaaaaagaactgatctgcattggactcgtaccacggttg-3′ |
| D158A | For | 5′-gtggtacgagtccaataaggcacagttctttttcctatatc-3′ |
|  | Rev | 5′-gatataggaaaaagaactgtgccttattggactcgtaccac-3′ |

^1^ For: sense primer and Rev: antisense primer; ^2^ blue bases correspond to modified codons and red ones correspond to insertions of a new codon.

**Table S3.** Sequences of primers used for quantification of PEMV1 and 2, and of transcripts for two housekeeping genes.

| **Accession No.** | **Description** | **Primer Name ^1^** | **Primer Sequence (5′-3′)** |
| --- | --- | --- | --- |
| X54844 (*P. sativum*) | β-tubulin | For-qPCR-Tub | TTCAGAACAAGAACTCGTCA |
|  |  | Rev-qPCR-Tub | CTCACCCTGCGGAACATTTCTTG |
| U76193 (*P. sativum*) | Actin | For-qPCR-Act2 | TGTCTTGGATTCTGGTGATGG |
|  |  | Rev-qPCR-Act2 | AGTTAGATCACGACCAGCAAG |
| NC003629 | PEMV1 | For-qPCR-V1 | GATTAAGCGCACAACTGAGGC |
|  |  | Rev-qPCR-V1 | GCTTGACCTTTGAGTTTC |
| NC003853 | PEMV2 | For-qPCR-V2 | AGGGTTTAAGATGAAGGTGGAAC |
|  |  | Rev-qPCR-V2 | AGATAGGCTGCGTTTGGC |

^1^ For: sense primer and Rev: antisense primer.


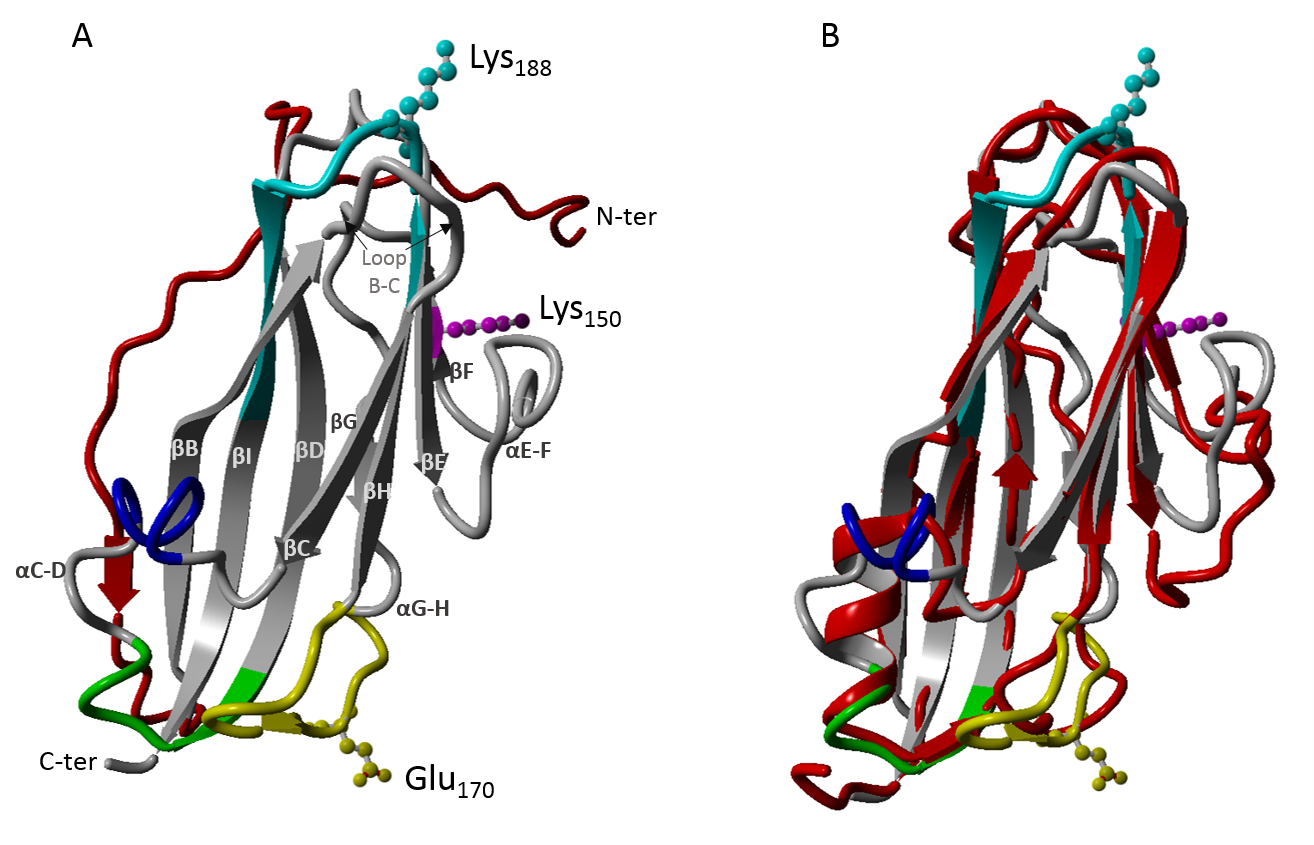


**Figure S1.** Predicted three-dimensional (3D) structure of *Potato leafroll virus* (PLRV) coat protein (CP) and comparison *to Pea enation mosaic virus (*PEMV) CP. (**A**) The R-domain is shown in red and the S-domain in grey. The PLRV S-domain epitope 10 residues in the loop regions between strands G and H are shown in yellow; epitope 5 is shown in blue. An acidic patch between strands C and D is shown in green (Lee et al., 2005). The peptide on the external surface of the virion that interacts with RTP is shown in cyan (Chavez et al., 2013). The residues implicated in protein–protein interactions are highlighted as follows: K188 (cyan; Chavez et al., 2013), K150 (Pink; DeBlasio et al., 2015), E170 (Yellow; Lee et al., 2005). (**B**) Overlay of the PLRV CP S-domain (mostly in gray) with the predicted structure of PEMV CP (red).


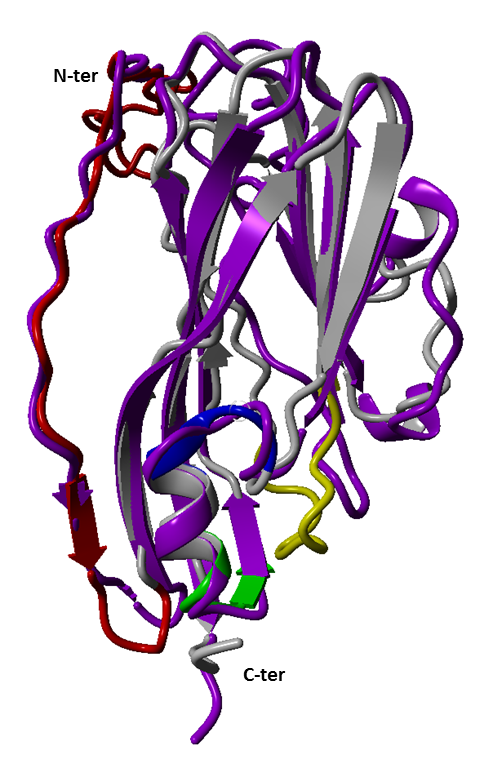


**Figure S2.** Overlay of predicted 3D structures of PEMV1 coat protein (CP) using Phyre2 and I-tasser servers. PEMV1 CP predicted structure obtained by Phyre2 is shown in purple. The one predicted by I-tasser has the R-domain in red and the S-domain in gray as described in Figures 1 and S1.


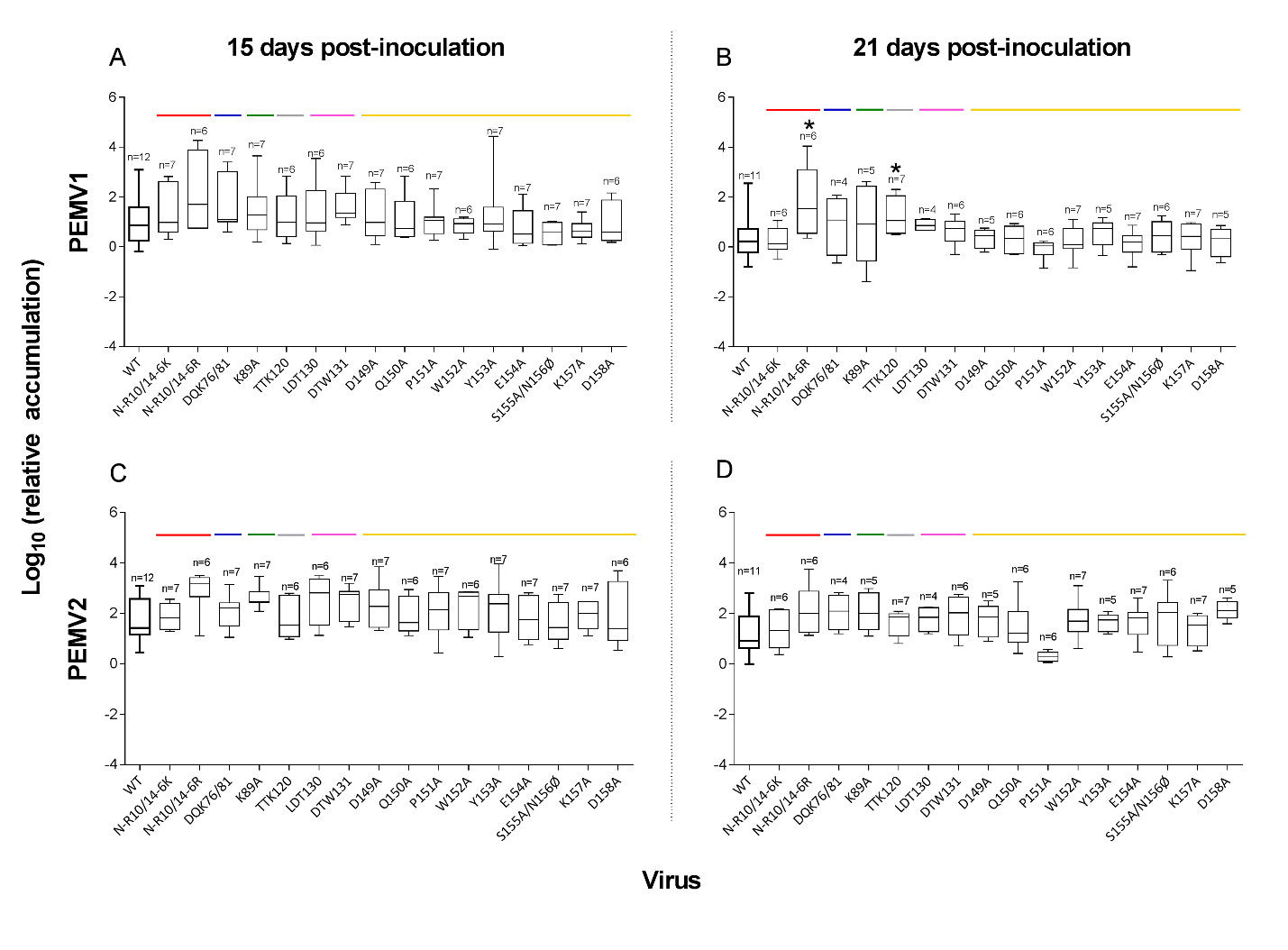


**Figure S3.** Accumulation of wild-type and mutant PEMV within inoculated leaves relative to
β-tubulin reference. (**A**,**B**) PEMV1; (**C**,**D**) PEMV2, WT and mutant accumulation at 15 and 21 days post-inoculation (dpi) of pea leaves, respectively. Virus accumulation was determined by RT-qPCR with reference to β-tubulin. The horizontal line within each box indicates the median value (50% quantile), while the box delimits the 25% and 75% quantiles. Colored lines correspond to the location of each mutant in the specific coat protein domains, as depicted in Figure 1A. The number (*n*) of infected leaves tested for each virus is indicated, with significant differences relative to the WT shown (* *p* < 0.05; ** *p* < 0.01).





**Figure S4.** Accumulation of wild-type and mutant PEMV within upper leaves of inoculated pea plants relative to β-tubulin reference. (**A**,**B**) PEMV1 and (**C**,**D**) PEMV2 RNA accumulation in upper leaves at 15 and 21 dpi of lower leaves. Viral RNA was quantified with real-time RT-PCR using β-tubulin as a reference. Colored lines correspond to the location of each mutant in the coat protein, as shown in Figure 1A. The numbers of infected leaves test for each virus are shown, with significant differences relative to the WT indicated (* *p* < 0.05 and ** *p* < 0.01).

| 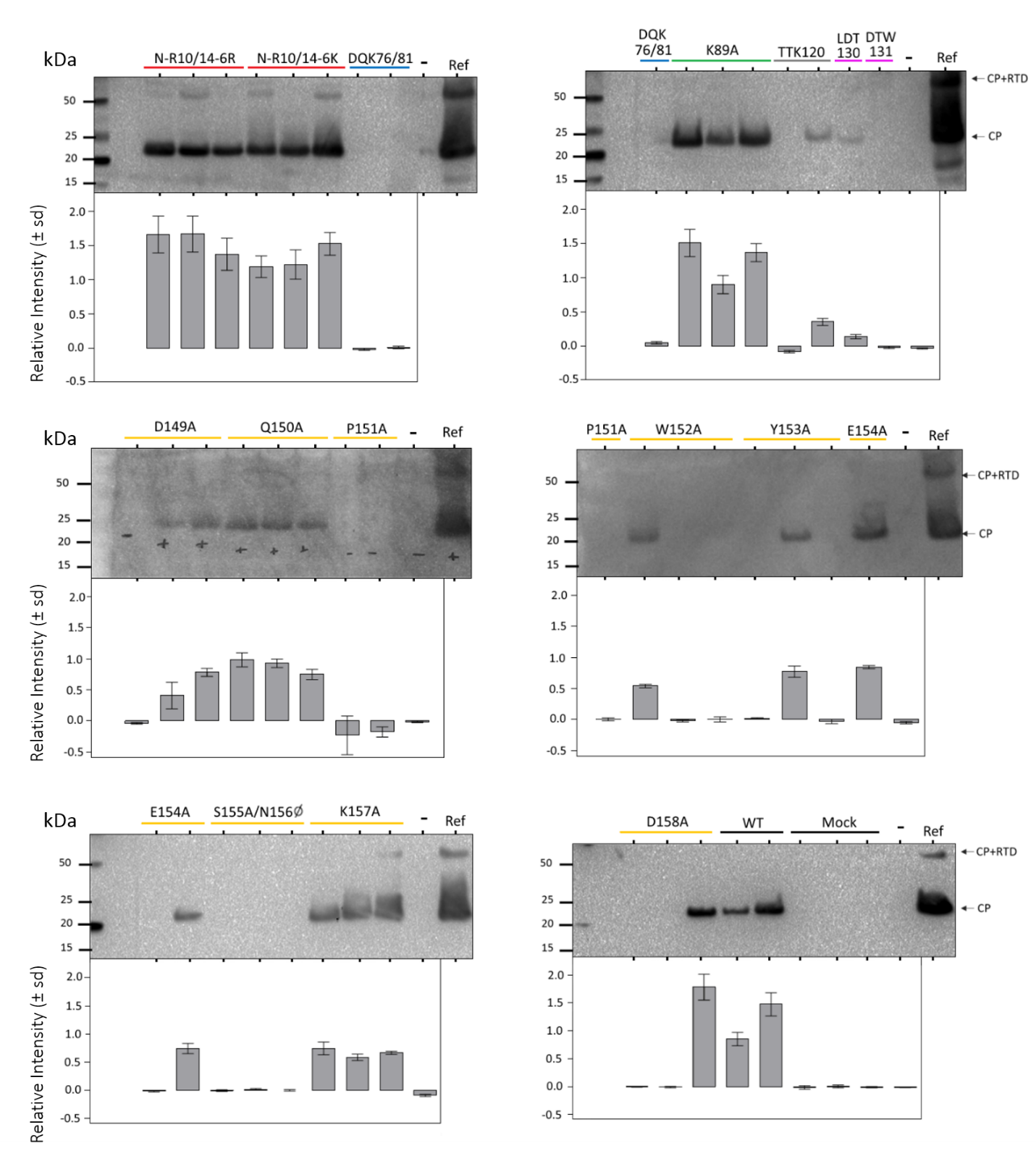 |
| --- |
| 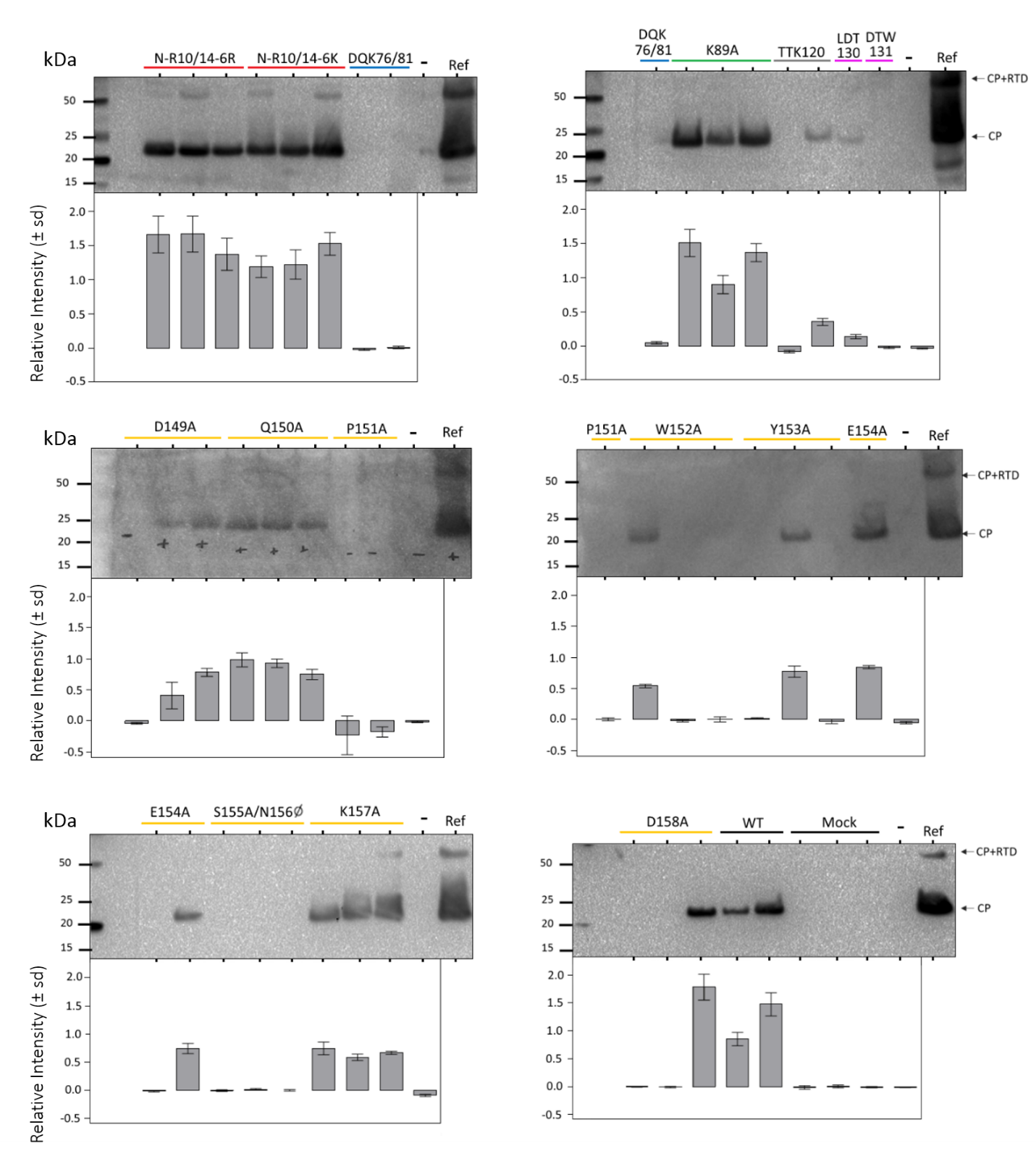 |

**Figure S5.** Detection of virion structural proteins following aphid inoculation of pea plants with
wild-type or CP mutants by western blot. Western blot images (Upper) were scanned and processed using GelQuant to estimate the relative amount of CP (histogram below*)* to a positive control. Only the CP mutant Q150A was efficiently transmitted by aphids. Leaves from plants infected with WT PEMV and uninfected leaves were used as positive and negative controls, respectively. Colored lines indicate the region in the CP for each mutation, as indicated in Figure 1A.

© 2016 by the authors. Submitted for possible open access publication under the
terms and conditions of the Creative Commons Attribution (CC-BY) license (http://creativecommons.org/licenses/by/4.0/).
